# Artificial Intelligence as a Tool in Diagnosing Inflammatory Bowel Disease in Older Adults

**DOI:** 10.3390/jcm14041360

**Published:** 2025-02-18

**Authors:** Ana-Gabriela Prada, Tudor Stroie, Rucsandra-Ilinca Diculescu, George Cristian Gogîrlă, Codruța Delia Radu, Doina Istratescu, Gabriel Ioan Prada, Mihai Mircea Diculescu

**Affiliations:** 1 Faculty of Medicine, “Carol Davila” University of Medicine and Pharmacy Bucharest, Bucharest 050474, Romania; ana-gabriela.prada@drd.umfcd.ro (A.-G.P.); rucsandra-ilinca.diculescu@drd.umfcd.ro (R.-I.D.); doina.proca08@gmail.com (D.I.); mmdiculescu@yahoo.com (M.M.D.); 2 Institutul Clinic FUNDENI Bucuresti (Fundeni Clinical Institute Bucharest), Bucharest 077086, Romania; gogirlageorgecristian@yahoo.com (G.C.G.); codrutaradu5@yahoo.com (C.D.R.); 3 Institutul Naţional de Gerontologie și Geriatrie “Ana Aslan” Bucuresti (“Ana Aslan” National Institute of Gerontology and Geriatrics), Bucharest 011241, Romania

**Keywords:** inflammatory bowel disease, older adults, diagnostic methods, artificial intelligence

## Abstract

**Background/Objectives**: The primary objective of our study was to find a potential use for images generated by imagistic investigations by comparing the appearance of a healthy digestive tract to that of a pathological one. **Methods**: We conducted a cross-sectional observational study involving 60 older adult patients admitted to and followed up at a primary center in Romania. Our focus was on different diagnostic methods and the use of artificial intelligence (AI) tools integrated into the electronic health records system. **Results**: Currently, imagery, laboratory values and electronic health records (EHR) can also be used to train AI models. Comparative imagery to predict the appearance of inflammatory bowel disease (IBD) can be used as a predictor model. **Conclusions**: Our findings indicate with certainty that training a tool in the diagnosis and prevention of relapses in older adults with IBD is promising for further integrating these models into patient care.

## 1. Introduction

Crohn’s disease (CD) and ulcerative colitis (UC) represent inflammatory bowel diseases (IBD) and are complex pathologies with different histological and imagistic presentations [1]. The clinical aspects of IBD in older adults, who are defined as individuals aged 65 and older in Romania, are frequently distinct from younger adults and often characterized by a largely silent disease [2,3]. The older adult population is also characterized as a multimorbid population, which restricts the possibilities of diagnostic methods [4] and sometimes delays diagnosis. Studying the particularities of this population can emphasize the challenges encountered by healthcare professionals when faced with an older adult patient who exhibits symptoms or laboratory findings consistent with IBD.

Challenges in the diagnosis of different pathologies in older adults are very common and sometimes impede finding an accurate diagnostic tool for this population [5]. Nowadays, we are discovering more sensitive and less invasive methods that will have a positive impact on older adults, as well as younger adults. Several studies by Alloca et al., Bots et al. and Zhang et al. [6,7,8] have shown that abdominal ultrasonography is useful in screening flare-up episodes in patients with IBD, thus limiting the need for supplementary invasive procedures.

Artificial intelligence (AI) is an emerging tool that is finding its way into aiding medical professionals to save time when screening pathologies. This new tool might help patients not yet diagnosed with IBD to avoid unnecessary, repeated, and uncomfortable investigations [9]. In some Western European countries, AI is used to interpret images and detect possible anomalies, but there is still a long way to go in developing a highly sensitive and accurate tool, due to ethical and data privacy considerations [10]. While the ethics of these methods are still debated [11], an important factor in the use of AI is the possibility to decrease the number of painful and uncomfortable investigations performed during the diagnosis and follow-up of these patients [12].

According to the FDA’s categorization of AI/ML-based medical devices, there is an AI-driven software function incorporated into an imaging system to aid healthcare providers in capturing ultrasound images of the shoulder region in both adult and pediatric patients. It enhances the imaging process by highlighting areas where a potential abnormality is detected in real time. It connects with the device acquisition system, processes its output using an AI model, and alerts the operator immediately if an abnormality is identified. It also automatically adjusts acquisition parameters to optimize imaging, but the device does not provide a diagnosis [13].

Another guideline that has been constituted by the World Health Organization (WHO) defines the uses of AI in medical devices [14].

After carefully studying the literature, we have identified a need to conduct a study on the diagnosis technique tendencies for older adults with IBD and the use of AI in the software of the devices used [15]. Older adults have more contraindications for different imaging techniques than young adults. Considerations include renal function, low tolerance to long examination times in patients with cognitive disturbances, and comorbidities that may limit the use of certain diagnostic methods.

Being an autoimmune disease, IBD is considered to result in important complications and a difficult clinical course for older adults. The literature [16,17,18,19] has shown that the diagnosis of older adults is more difficult because of silent symptomatology and the geographical distribution of patients. A study on vitamin D and cognitive status has highlighted the difficulty of differential diagnosis in this population [20].

A few studies have shown that CD with onset after the age of 65 years is more frequently associated with isolated colonic inflammation and perianal fistulas, while small bowel and upper gastrointestinal diseases are more rarely seen [21,22,23]. Older adults diagnosed with UC more often exhibit left-sided and extensive involvement of the colon, while isolated proctitis is less commonly encountered and only a small fraction of patients show extension of the lesions at follow-up [22,23]. Studies have shown that there is a 6-year delay in the diagnosis of IBD in older adults compared to younger adults [23,24]. Moreover, older adults show a more silent clinical presentation of CD and UC, with less abdominal pain, diarrhea, and rectal bleeding [1,24].

Differential diagnosis is needed in older adults with IBD to exclude other pathologies that can mimic the clinical presentation of these pathologies. Thus, the exclusion of malignancies, infectious diseases, diverticulitis, ischemic colitis, microscopic colitis, drug-induced colitis, and radiation-induced colitis is crucial in the timely diagnosis and treatment of the underlying pathology [23,25,26].

A recent study conducted in Poland on 47 patients with IBD [27] concluded that the chondroitin sulfate fraction can be quantitatively assessed through urine samples in patients with UC to monitor the effectiveness of adalimumab treatment. This study [27] presents further research on the remodeling of the extracellular matrix components theory that is responsible for the changes in the proteoglycans and glycosaminoglycans that can be seen at the molecular level in IBD patients [28].

The AWARE-IBD Diagnostic Delay Working Group has recently published an interview-based study where the key points were related to key individuals involved in the delay of patient diagnosis, highlighting that patients themselves, generalists, and specialists all contribute to delays in the diagnosis of CD and UC. The working group also emphasized that the diagnosis process and clinical experience should take into consideration the atypical and silent characteristics of IBD in older adults [17].

A study published in 2020 found that epidemiological studies highlight a particularity of IBD in older adults that the most affected segment of the gastrointestinal tract was the colon, but the course of the disease was milder and extraintestinal manifestations were less common. The same study refers to the increased mortality of this population due to comorbidities, polypharmacy, and reduced resistance to severe disease [23].

In Romania, artificial intelligence has existed for decades, but in recent years, it is slowly becoming a useful tool in healthcare as well as other domains. The COVID-19 pandemic has encouraged the rapid digitalization of health services, with telemedicine helping doctors keep in contact with isolated or frail patients [29].

Pattern recognition has been an established practice in areas such as weather forecasting [30], trading [31], and drug development [32]. Now, with increased availability, more focus, and decreased prices for generating Large Language Models (LLM), disciplines like healthcare must adapt and utilize this new framework as well. Because there is an existing database, LLM can be trained to find similarities in the patterns seen before reaching a probable diagnosis. Guidelines are readily available online, so by comparing data found in the guidelines with data related to a patient’s symptoms and laboratory results, LLM can aid in the timely diagnosis of certain pathologies, thus contributing to a decreased prevalence of complications and possibly shorter hospital stays [33].

One study showed a 20% increase in bone fracture detection by using a trained model that has had its accuracy, sensitivity, and specificity in detecting fractures evaluated by comparing its predictions with existing images that have been accepted as correct [34].

A study performed in the United States has shown that in the future, it is expected that the quality of pathophysiological descriptions of diseases will become more accurate with the help of AI, enabling more personalized medical care, but it will be used as an integral part of clinical practice as an unbiased tool rather than taking control of diagnostic and treatment approaches [35].

The results of our study provide insight into the further development of AI-based software that will be able to analyze and predict IBD and related relapses, while using the most effective and least invasive diagnosis methods and follow-up investigations. This will have a positive impact on the quality of life of older adults with IBD, and it will aid in developing more conscious treatment plans with the help of an advanced tool.

## 2. Materials and Methods

### 2.1. Study Type

**Figure 1:**
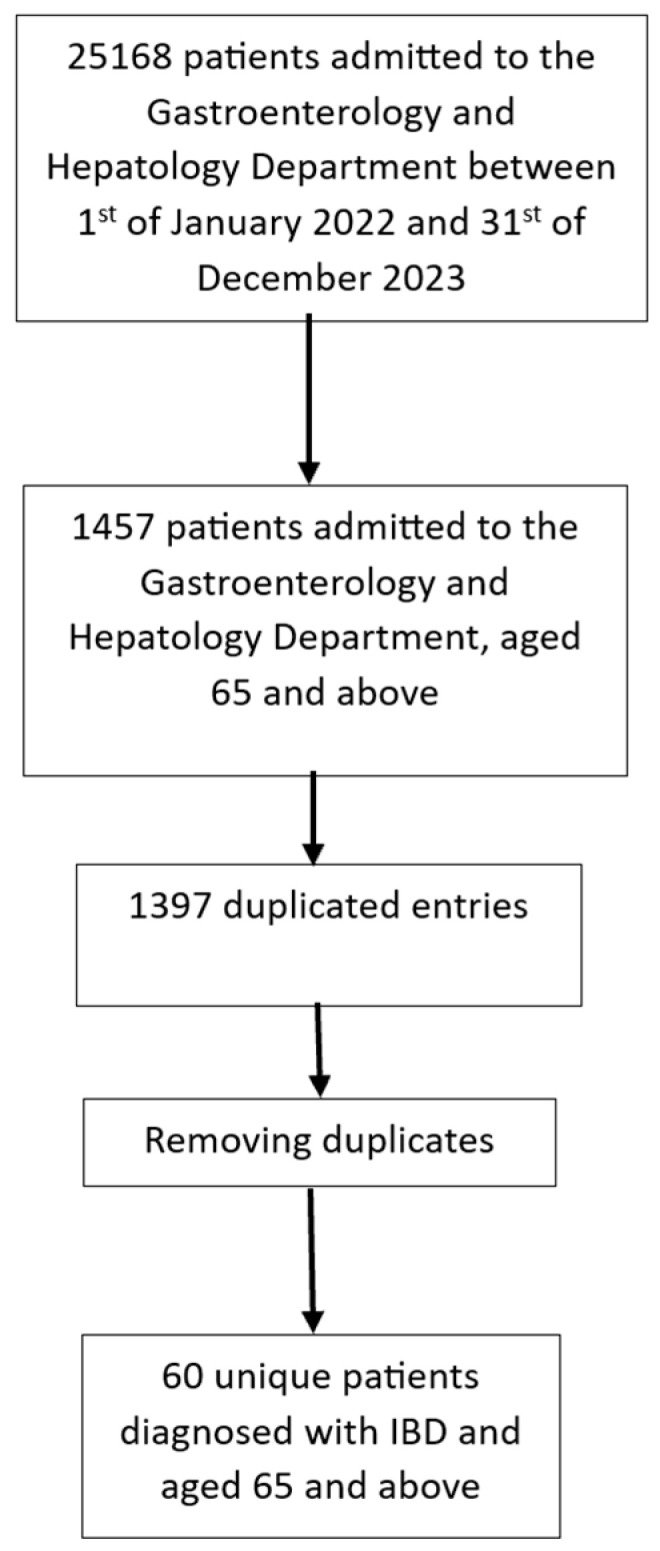
Sorting algorithm for patient database.

We have conducted a cross-sectional observational study by accessing the electronic records of patients admitted to the clinic between 2022 and 2023. The data were searched for by applying filters and sorting algorithms according to the age of the patient, International Classification of Diseases 10th Revision (ICD-10) diagnostic codes, the department they were admitted to, and the period of admission to the department (between 1 January 2022 and 31 December 2023). Without filtering out the number of presentations to the department and including patients at the age of 65 years or older at the time of admission, we obtained a database of 1457 patients. Filtering of the dataset was carried out in multiple stages. We synthesized the data by removing duplicates because our study focused on individual cases, not numbers. We included 60 unique patients out of the whole database. These patients were followed up in the Departments of Gastroenterology at the Clinical Institute Fundeni (ICF), a primary healthcare center in Bucharest, Romania, and we compared our results to findings in the literature. Figure 1 depicts our sorting algorithm that concluded with the database used for this study.

We searched for AI tools used in diagnosis and compared them to the availability of AI-based imagistic investigations at re-evaluation. We hypothesized that machine learning can be helpful in the early diagnosis of IBD in older adults because it makes predictions based on the data it is based on.

We excluded patients who did not have a diagnosis of IBD, people aged less than 65 years, and patients that did not agree to take part in the study.

After alphabetical sorting of the database, multiple presentations of the same patient were identified and excluded from the quantitative analysis of the database.

We have represented our dataset characteristics in Table 1 to better understand the distribution of the diagnosis methods and the use of AI across age groups, gender and IBD type.

### 2.2. Ethical Aspects

All patients signed an informed consent form upon admission to the hospital regarding inclusion in this study. The patients raised ethical issues when asked if they would trust an automated system to search for pathological findings on images and expressed a fear of data leakage. The use of AI was met with caution by the older adults included in the study, thus limiting the use of AI tools in the diagnosis of IBD in this population. The data controller has sole responsibility for the data and the data processor uses the data but cannot be liable for data leakage. Therefore, the data controller can be the company that produces the computer vision tool and has full control over the information input and output.

### 2.3. Digital Systems Used

With the help of the “Hipocrate” and “InfoWorld” digital systems, more and more hospitals in Bucharest and Romania can use an electronic database and have a backup of files for each patient. Access to these files is available to all medical personnel by introducing a username and a password. A shared electronic health record (EHR) has helped enormously in sharing research data, as well as organizing and providing easy access to this information, while adhering to ethical principles and ensuring the protection of data.

### 2.4. Statistical Analysis

The data retrieved from the patients’ electronic records were introduced into a Microsoft Office Excel worksheet, sorted alphabetically, and then analyzed using the analysis functions included in the program.

The results were represented graphically with the help of the statistical analysis conducted using IBM SPSS version 30.0.0 to perform descriptive analysis (frequencies, descriptives and population descriptives) and power analysis (to test for means by using one-way simple *t*-test).

In our study, we considered an AI tool as a computer vision AI, meaning that the computer can read an image. We have considered this tool as a machine learning tool and not as generative AI, because it was used to enable the analysis of images and not to generate images or diagnoses. This means that this tool was considered an aid for diagnosis and not a definitive diagnosis method.

## 3. Results

Studying the characteristics of the older adult population with the use of new methods poses ethical as well as cultural challenges. Therefore, we informed study participants that the imagistic investigations would comply with data protection laws and that no information would be linked directly to any individual.

After careful analysis of the data gathered from our patients’ history and investigations, we obtained valuable information related to how the distribution of older adult patients with IBD reflects the clinical characteristics of these patients. Gender plays an important role in studying clinical outcomes and possible hormonal influences on the frequency of relapses and treatment adverse effects.

We grouped the methods of diagnosis according to the year of diagnosis so that we could identify which methods had access to the use of AI at that moment.

Because most diagnostic methods are imagistic and measurements are attached to the images, AI can be seen as a useful tool for reducing examination time and guiding toward a more patient-targeted treatment course.

Laboratory investigation values are another variable that can be included in building the AI model, which will aid in the prediction and possibly prevention of relapses and complications. These findings can also aid in timelier scheduling of follow-up when looking at the pattern of increase in inflammatory markers and specific laboratory investigations (calprotectin).

In this study, we mainly focus on imagistic investigations because, presently, they are the only ones with available AI input on key findings that need to further be interpreted by a specialist. We are hoping to develop software for laboratory investigations that will integrate the use of AI in the future.

### 3.1. Diagnosis Methods Used in Older Adults with IBD That Had an AI-Assisted Preliminary Results Interpretation

**Figure 2:**
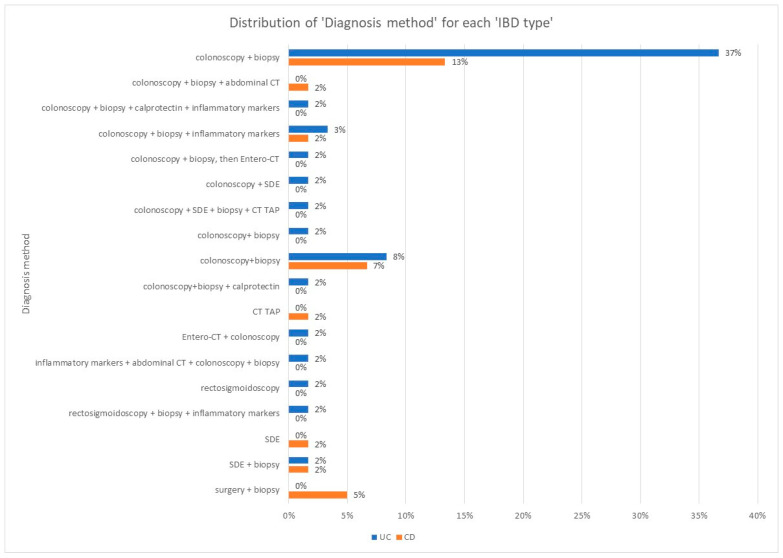
Distribution by IBD type of most commonly used diagnosis methods in older adults.

The data represented in Figure 2 show that only three types of diagnostic approaches are used in the case of CD, the most frequently used being colonoscopy with biopsy. In the case of UC, colonoscopy with biopsy is the most used, but in the case of this condition, six other diagnostic methods are also used: rectosigmoidoscopy, superior digestive endoscopy, colonoscopy with entero-CT, colonoscopy with biopsy and deep sedation, Pansdorf, and colonoscopy with CT TAP. The difference between the prevalence of using colonoscopy with biopsy for UC and CD is highly statistically significant (*p* = 0.0007, chi-square = 11.503), with a higher use in UC.

We note that the only diagnostic methods used in both types of pathology are colonoscopy with biopsy (the most frequent) and rectosigmoidoscopy, the latter having a quasi-similar prevalence between 3% and 4%.

**Figure 3:**
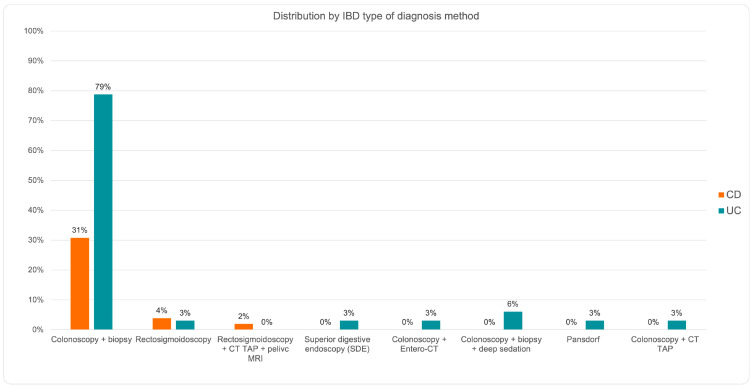
Distribution by IBD type and diagnosis methods in older adult patients.

As can be seen from Figure 3, 79% of the older adult patients diagnosed using colonoscopy with biopsy in the Gastroenterology and Hepatology Clinics of the ICF were diagnosed with UC, and 31% of all patients diagnosed using the same method had CD. Among patients diagnosed with CD using other diagnostic methods, rectosigmoidoscopy was the most commonly used (4% of patients with CD), followed by rectosigmoidoscopy supplemented by CT TAP (2% of these patients).

Older adult patients diagnosed with UC benefited from several types of diagnostic methods, including rectosigmoidoscopy (3%), EDS (3%), colonoscopy supplemented with entero-CT (3%), colonoscopy supplemented with biopsy and deep sedation (6%), Pansdorf (3%), and TAP CT colonoscopy (3%).

Rectosigmoidoscopy with TAP CT and pelvic MRI was not used for patients with UC, either because other methods provide enough data for a positive diagnosis, or because this investigative combination is laborious and expensive, with the benefits for the older adult patient not being sufficient enough.

To stratify the diagnostic methods used for patients with IBD according to age group, it is necessary to study the distribution of patients with IBD by age group, regardless of the diagnostic method used. This distribution contributes to a thorough understanding of patient type in the ICF Gastroenterology Department and the challenges associated with these patients’ comorbidities. Thus, we agreed to categorize IBD patients admitted to the ICF Gastroenterology and Hepatology Clinic by the following age groups: 65–69 years, 70–74 years, 75–79 years, 80–84 years, and 85 years and over.

**Figure 4:**
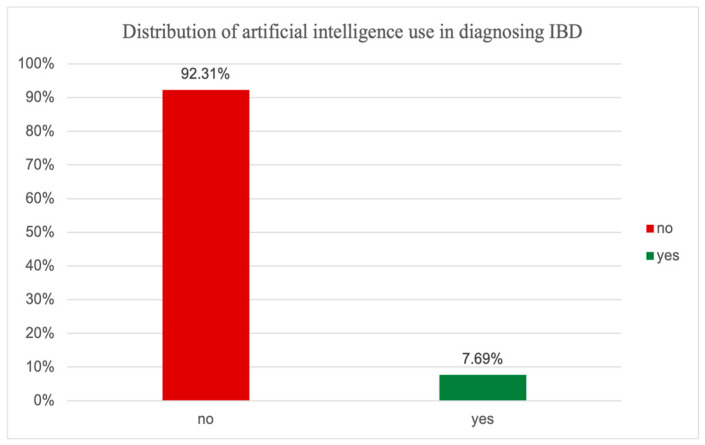
Distribution of artificial intelligence use in diagnosing IBD in older adults.

Figure 4 shows that 92.31% were diagnosed with methods that did not use AI, while only 7.69% were diagnosed through methods that used AI. This indicates that AI is not widely used in IBD diagnosis, at least among the population included in our study. This shows that for imagistic diagnosis, healthcare professionals do not rely on the use of AI-powered software and prefer conventional methods that base results on the experience of the examiner and the quality of the preparation for the examination (for example, for colonoscopy and superior digestive tract endoscopy).

### 3.2. Diagnosis Methods Used in Different Age Groups

**Figure 5:**
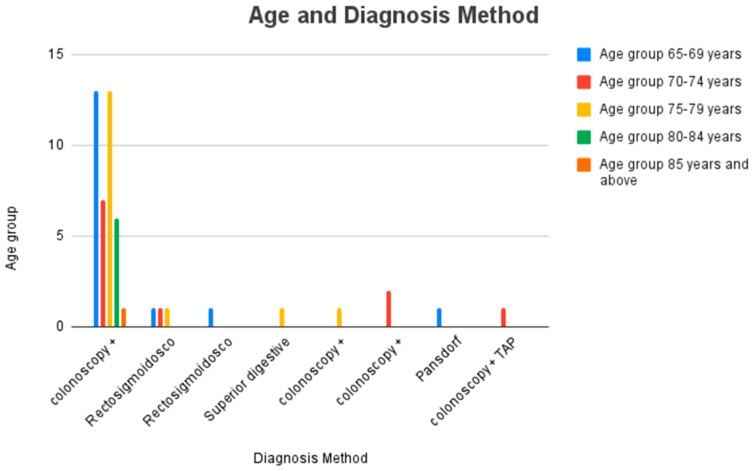
Distribution by age group and diagnosis method used for older adult patients with IBD.

Given that in the 1980s and 1990s the preferred diagnostic method for the diagnosis of IBD was colonoscopy with biopsy, a preference for using colonoscopy with biopsy to diagnose all patients, regardless of current age, can be seen in Figure 5.

The lower prevalence of the use of colonoscopy with biopsy in patients aged 70–74 years (64% of this age group) highlights a need to use rectosigmoidoscopy and colonoscopy accompanied by CT TAP for the diagnosis of IBD in this population group.

For patients aged 85 years and older and for those aged 80–84 years, the only diagnostic method used was colonoscopy with biopsy.

Figure 4 highlights that diagnostic methods are highly diversified for patients in the 65–69 age group, with the highest prevalence for diagnosis by colonoscopy with biopsy (81% of the patient group), with a distribution of 6% for the following methods: rectosigmoidoscopy, the Pansdorf method and rectosigmoidoscopy with CT TAP and pelvic MRI.

The difference between the prevalence of the use of colonoscopy with biopsy in patients aged 65–69 years and the use of the three previously mentioned diagnostic methods for the same patients is highly statistically significant (*p* = 0.0391, chi-square = 4.254) in favor of diagnosis by colonoscopy with biopsy in the 65–69 age group.

### 3.3. Differences in Diagnosis of Male and Female Older Adults with IBD

**Figure 6:**
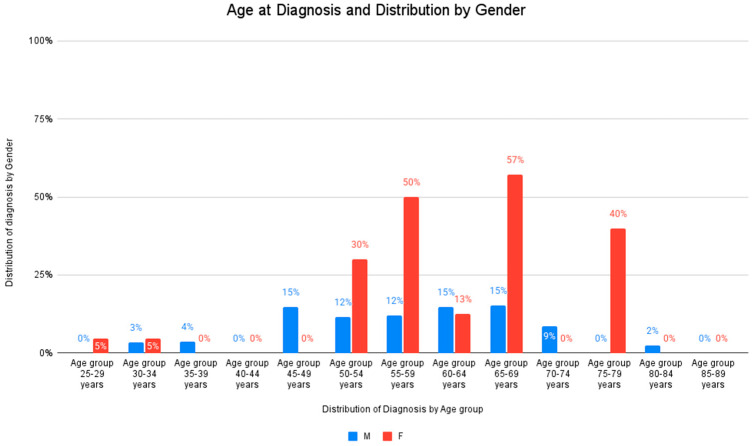
Distribution by gender and age at diagnosis of older adult patients with IBD.

The graph shown in Figure 6 presents a distribution of the studied patient population by age at diagnosis and gender, with a focus on the percentage of males (M) and females (F) in various age groups. The age groups are categorized into intervals ranging from 25–29 years to 85–89 years, while the percentage of patients within each age group diagnosed is divided by gender. The blue bars represent the percentage of males diagnosed with IBD within each age group, while the red bars represent the percentage of females diagnosed with IBD within each age group, with very low percentages (0–5%) of both males and females diagnosed in the younger age groups (25–44 years) and no males diagnosed in the 25–29 years and 35–44 years age groups.

Table 2 highlights the difference in diagnosis rates between males and females, with a mean difference of 0.45 between genders and a *t*-test value of 6.948 (95% CI = 0.32–0.58).

A noticeable increase in diagnoses appears in the age group 45–49 years, particularly for males (16%) and also for females (8%). Females in the age group 55–59 years have a significant increase of 50%, compared to 13% in males. The trend continues with an even higher percentage of females being diagnosed, especially in the 65–69 years age group, where 57% of females are diagnosed, compared to only 14% of males. A similar trend is seen in the 60–64 and 70–74 age groups, where females have higher diagnosis rates compared to males.

For the age group 75–79 years, only females are represented, with a 50% diagnosis rate, while no males are diagnosed. The 80–84 age group shows a small percentage of males (2%) diagnosed, with no females represented, while no patients were diagnosed for either gender in the 85–89 years age group.

The blue dotted line in Figure 6 shows a relatively flat trend for male diagnoses across age groups, indicating little variation across ages. The trendline for females, represented by the red dotted line in the graph, shows a slight upward slope, indicating that the likelihood of diagnosis increases slightly with age for females.

The R^2^ value for males (0.0008) suggests that the trendline is not a good predictor of male diagnosis rates, while the R^2^ value for females (0.0567) indicates a slightly better, though still weak, correlation between age and diagnosis rate for females.

Thus, the graph in Figure 5 emphasizes that females have a higher diagnosis rate compared to males, particularly in the middle-to-older age groups (55–74 years), and the likelihood of being diagnosed increases with age, particularly for females, while the diagnosis rate for males shows minimal change across different age groups.

## 4. Discussion

Colonoscopy with biopsy is the overwhelmingly preferred method for diagnosis, used in 81% of cases. This suggests it is considered the gold standard or most reliable method for diagnosis in this context. The wide disparity in usage indicates that while there are multiple methods available, colonoscopy with biopsy is the dominant choice, likely due to its effectiveness and current standard practice guidelines.

Our study group showed an increased prevalence in the diagnosis of IBD in older adult patients, especially in the male population (57.7%), which agrees with the specialized literature [16,36] but contrasts with the higher prevalence in people of the female sex in the Romanian population [37].

Our studies showed an increased prevalence for diagnosing older adult patients using colonoscopy with biopsy, regardless of the type of IBD identified, which is also found in the specialized literature to be a particularity of the diagnostic method used in this population group.

A diagnostic method that has been noted among those previously mentioned is the Pansdorf method, also known as barite transit, which represents an outdated diagnostic modality with low specificity and sensitivity for IBD. Considering these characteristics of the previously mentioned method, the standard for the diagnosis of IBD remains to be colonoscopy with biopsy [38].

Of the study group, 79% of patients with UC and 31% of patients with CD were diagnosed by colonoscopy with biopsy. The difference between the prevalence of using colonoscopy with biopsy for UC and CD is highly statistically significant (*p* = 0.0007, chi-square = 11.503) in favor of UC.

Since the 1980s, colonoscopy with biopsy has been the standard diagnostic method for IBD in both young and older adult patients. It should be emphasized that in the 2020s, this diagnostic method is still widely used, with a prevalence of 73% of the group of diagnosed patients, compared to 9% for the other three diagnostic methods. The difference between these two prevalences is highly statistically significant (*p* = 0.0540, chi-square = 3.714) in favor of colonoscopy with biopsy.

Populational studies in Romania [3] have shown that there is a higher percentage of older adults in rural areas, which could explain the easier access to diagnostic methods that use AI and LLM to predict relapse episodes.

The distribution suggests that IBD is mostly diagnosed in individuals aged 65–69 years and 75–79 years, with each of these groups making up 32% of the patient population. The 70–74 years age group also has a significant portion (22%) of patients. There is a noticeable decline in the prevalence of IBD in patients older than 80 years, especially in those aged 85 years and above, which only account for 2% of the patients.

This new tool could potentially be of great value based on results from other studies [9,11,12,34,35]. Models like this become better and better, but they need to obtain data that show what a wrong answer is and what a correct answer is, which will take time, and a definite diagnosis will still need a physician’s approval and input.

The limitations of our study are related to small cohort research, the character of single-center research, and the limited use of AI in diagnosing IBD.

In future work, we will conduct cross-sectional and prospective studies in which we will also analyze other factors that can influence the early diagnosis of this type of gastrointestinal disease, as well as therapeutic management strategies according to various specific parameters of the patients.

The use of AI in the preliminary results of imagistic investigations can aid in a more targeted approach [9] to the older adult patient with IBD, and with recent advancements and curation of data by AI, the diagnosis of older adults with IBD will be less invasive and performed sooner.

Even though there were not enough diagnosis methods that used AI to aid in discovering the presence of IBD in older adults in this study, with the improvement of AI and the discovery of patterns in the diagnosis of IBD, this new tool will be of great help in the future.

The small number of people included in our study group limits the possibility of further discussing the use of AI in all older adults with IBD, but it is of great interest to our department to develop tools that use AI.

## 5. Conclusions

Even though AI is a new and evolving tool which aids in other fields, it is still underused in medicine and diagnostics. Future training models and an extensive database will contribute greatly to the better care of adults and may help predict disease onset and relapse episodes earlier.

## Figures and Tables

**Table 1:** Descriptive analysis of dataset.

Ulcerative colitis patients	67%
Crohn’s disease patients	33%
Rural residents	13%
Urban residents	87%
Male patients	55%
Female patients	45%

**Table 2:** One-sample *t*-test regarding difference in diagnosis rates between male and female older adult patients.

One-Sample Test							
Test Value = 0							
t		df	Significance		Mean Difference	95% Confidence Interval of the Difference	
			One-Sided *p*	Two-Sided *p*		Lower	Upper
Gender	6.948	59	<0.001	<0.001	0.45	0.32	0.58
IBD type	10.863	59	<0.001	<0.001	0.667	0.54	0.79

## Data Availability

Data is available upon reasonable request.
